# The Prevalence of Postpartum Depression and the Related Risk Factors in Primary Health Care, Al-Madinah, Saudi Arabia

**DOI:** 10.7759/cureus.22681

**Published:** 2022-02-28

**Authors:** Ruba Abdulqader Alrehaili, Reema Albelowi

**Affiliations:** 1 Family Medicine, Academy of Family Medicine, Ministry of Health, Al-Madinah, SAU

**Keywords:** postpartum, al-madinah, women's mental health, saudi women, postpartum depression

## Abstract

Background

The onset of depressed symptoms within six weeks of childbirth is described as postpartum depression (PPD). When compared to developed countries, resource-constrained countries have a higher prevalence of PPD. The purpose of this study was to determine the prevalence of PPD and the factors linked to it in primary health care in Al-Madinah, Saudi Arabia.

Methods

This descriptive cross-sectional study was carried out at the Academy of Family Medicine, Al-Madinah Almunawarah, between June 2021 and December 2021. The Arabic version of the validated Edinburg Postnatal Depression Scale was used to measure postpartum depression. SPSS version 26 (IBM Corp., Armonk, NY) was used to record and analyze the data.

Results

A total of 243 participants were included in this study. The mean age of the participants was 28.21±11.54 years. Most of the participants, 116(47.7%), were in the age group of 22-30 years. Most of the babies 176 (72.4%) were born via normal vaginal delivery (NVD) while only 67 (27.76%) were born via caesarian section. Most of the participants had an income of <5000 riyals in our study. In our study, the majority of the babies, 137 (56.4%), were breastfed. In our study, major depression was found in 77 (31.68%) participants while mild depression was present in 50 (20.57%) participants. The comparative analysis showed that previous children (p <0.001), support in caring (p = 0.02), income status (p = 0.003), and child health issues (p<0.001) were statistically significant.

Conclusion

The prevalence of postpartum depression was relatively high in our study. The number of prior children, child health, income status, and family support all have a positive association. In short, healthcare providers must pay special attention to the mental health needs of pregnant women in our settings.

## Introduction

The postpartum period has been linked to a higher chance of developing maternal depression. According to The Diagnostic and Statistical Manual of Mental Disorders, Fifth Edition, postpartum depression (PPD) is defined as a moderate-to-severe depressive episode that often begins within four weeks of birth. Anxiety, sadness, sleep disturbances, appetite disturbances, lack of energy, disorientation, guilt or worthlessness, and suicidal thoughts are all symptoms of postpartum depression. Postpartum depression can have a big impact on both the mother-infant bond and the child's development [1].

During the first month after giving birth, 40% of mothers experience maternal blues, which are generally mild, self-limiting, and have little impact on the mother and child [2]. However, if it is not detected and treated properly, the woman is more likely to develop postpartum depression. Postpartum depression affects 13% to 19% of mothers, and if not treated properly, it can lead to postpartum psychosis. PPD is a non-psychotic depression episode that affects moms between the ages of 4 and 6 months after giving birth [3].

PPD may have a significant impact on many parts of a mother's and child's lives. Close monitoring of moms at risk for PPD and referral when there is a substantial indication is one of the most critical roles of family doctors [4].

Postpartum depression risk factors must be investigated in order to diagnose and prevent postpartum depression early [5]. Psychological, biological, and social risk factors for PPD have been identified [6]. Women's ages, marital status, marriage ages, number of children, education status and occupations of women and their spouses, income levels, unintended pregnancy, type of feeding (breast, bottle, or both), lack of family support for baby care, negative life events, and poor night-sleeping patterns of babies are just a few of the things that have been found to be important.

Postpartum depression is a prevalent condition among women of childbearing age that not only affects moms but also entire families. Screening for emotional and psychological disorders, especially in those at risk, should be part of the follow-up. The goal of this study was to find out how common postpartum depression is and what variables contribute to it at a primary health care facility in Al-Madinah, Saudi Arabia.

## Materials and methods

This descriptive, cross-sectional research was done at the Academy of Family Medicine in Al-Madinah Al-Munawarah, which is located in northern Saudi Arabia, from July 2021 to December 2021. Women who had just given birth and were visiting well-baby clinics in primary health care for 30-45 days postpartum were included. Those that refused to participate were excluded. By using the World Health Organization (WHO) sample size formula: n=Z2 (1-a) P(1-P)/d^_2_^. The minimum sample size necessary was determined to be 243. The study was conducted in five primary health centers (PHC) in Al-Madinah, with one center from each cluster chosen at random and the total number of female patients attending the PHC in the previous month determined.

To assess postpartum depression, the Arabic version of the well-known and validated Edinburgh Postnatal Depression Scale (EPDS) questionnaire [7] was employed. The Edinburgh Postnatal Depression Scale has been translated and validated in Arabic [8], and it is extensively used throughout the Arab world. A cut-off score of 13 was seen as indicating likely depression. Ethical permission was granted by Madinah's regional research and ethics commission. Throughout the process, data confidentiality was ensured.

Data were analyzed using the Statistical Package for the Social Sciences version 26 (IBM Corp., Armonk, NY). Qualitative variables were presented as frequency and percent. Quantitative variables were tested for normality distribution and were presented as mean and standard deviation. The chi-square test was used for group comparison. This study considered variables statistically significant at p < 0.05.

## Results

A total of 243 participants were included in this study. The mean age of the participants was 28.21±11.54 years. Most of the participants, 116 (47.7%), were in the age group of 22-30 years, followed by 31-40 years, 72 (29.6%). The majority of the babies, 176 (72.2%), were born via normal vaginal delivery (NVD) while only 67 (27.76%) were born via cesarean section. Of our participants, only 112(46.1%) had planned pregnancies. Most of the participants had an income of <5000 riyals in our study. Table 1 shows the demographic data of the participants.

**Table 1:** Demographic characteristics of participants (n=243) NVD: normal vaginal delivery

Variable	Frequency	Percentage
Marital status		
Divorced	25	10.3%
Married	218	89.7%
Age group		
14-21 Years	31	12.8%
22-30 Years	116	47.7%
31-40 Years	72	29.6%
>40 Years	24	9.9%
Age of baby		
0 month	67	27.6
1 month	64	26.3
4 months	78	32.1
6 months	34	14.0
Mode of delivery		
Caesarean	67	27.6
NVD	176	72.4
Pregnancy planned		
No	131	53.9
yes	112	46.1
Family income		
<5000	91	37.4
>15000	25	10.3
10000 - 15000	44	18.1
5000 - 10000	83	34.2
Baby feeding		
Bottle	42	17.3
Beast	137	56.4
Mixed	64	26.3

**Figure 1:**
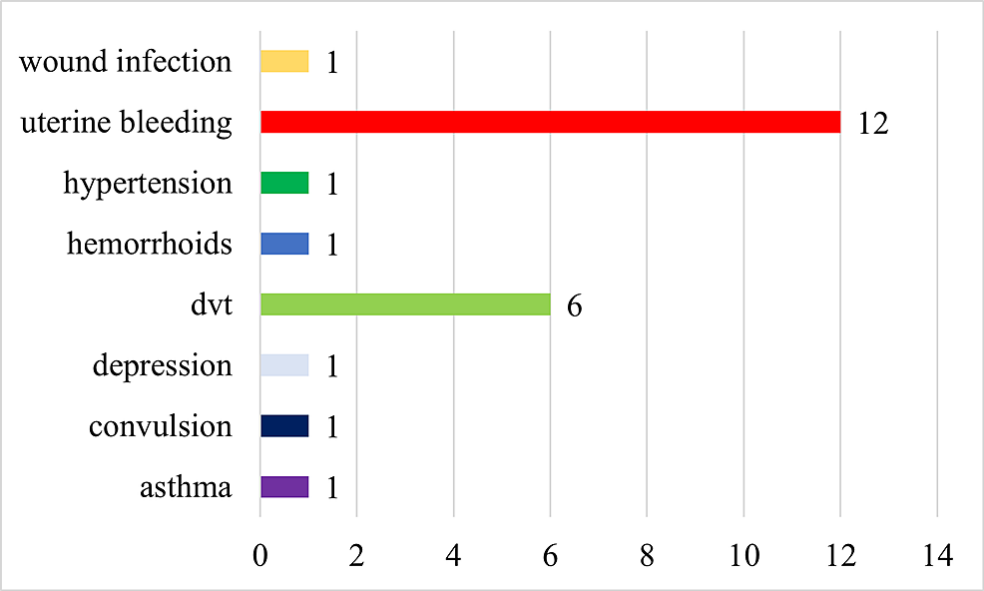
Complications of the participants in our study DVT: deep vein thrombosis

Regarding previous children, most of the participants, 113 (46.50%), had zero to two previous children, 85 (34.97%) had three to four children, and only 45 (18.51%) had five previous children at home. One-hundred-fifteen (115; 47.35%) of the participants were helped by family and friends in assisting the babies while the rest were doing it on their own. Of the participants, 9.87% had postpartum complications. Figure 1 shows the complications, out of which uterine bleeding was on the top (12; 4.93%).

Table 2 Shows the Edinburg Postnatal Depression Scale mean values.

**Table 2:** Mean score of Edinburgh Postnatal depression scale

Sn	Scale	Mean	SD
1	I was able to feel joy and happiness	0.76	0.73
2	I looked forward to things with pleasure	0.83	0.85
3	I blamed myself needlessly when things went wrong	0.96	0.91
4	I was worried and preoccupied for no good reason	1.07	0.955
5	I felt scared and panicked for no good reason	0.92	0.91
6	I had a lot of work, and I could not do it all	0.92	0.98
7	I was so unhappy that I had trouble sleeping	0.90	0.97
8	I felt unhappy and miserable	0.79	0.91
9	I was so unhappy and in so much pain that I cried	0.68	0.865
10	I had the idea of hurting myself	0.36	0.66

According to the findings, 77 (31.68%) had an EPDS score of 13 or higher, indicating likely depression, whereas 50 people (20.57%) had an EPDS score of 10-12, indicating potential depression. Only five ladies considered injuring themselves due to suicidal thoughts (EPDS, question 10). Postpartum depression was shown to be statistically linked with demographic characteristics such as prior children (p <0.001), help in caring (p 0.02), financial level (p 0.003), and child health difficulties (p <0.001). See Table 3.

**Table 3:** Comparative analysis of Edinburgh Postnatal Depression Scale with demographic data NVD: normal vaginal delivery; C/S: cesarean section

Variables	No depression	Mild depression	Major depression	P-value
Age				
14-21	16	2	13	0.122
22-30	61	26	29	
31-40	30	18	24	
>40	9	4	11	
Marital status				
Married	108	42	68	0.185
Divorced	8	8	9	
Previous children				
0-2	62	16	35	0.000
3-4	46	21	18	
5	8	13	24	
Support in caring				
No	52	27	50	0.023
Yes	64	23	27	
Income status				
<5000	30	22	39	0.003
>15000	9	7	9	
10000-15000	30	6	8	
5000-10000	47	15	21	
Mode of delivery				
NVD	88	34	54	0.502
C/S	28	16	23	
Child health issue				
Yes	0	9	10	0.000
No	116	41	67	
Baby feeding				
Bottle	13	14	15	0.37
Breast feed	74	26	37	
Mixed	29	10	25	

## Discussion

The prevalence of postpartum depression in mothers was found to be 31.68% in our study. The reported prevalence of PPD was greater than that reported in previous studies in western nations; for example, the prevalence of PPD in North America was 10%-15% and in Australia, it was 13% [9-10]. These statistics showed that the prevalence of PPD was higher in the Middle East than in other parts of the world. The predicted reasons for the wide range of reported PPD could be due to disorders and displacement during crises, cross-cultural variation, socio-economic conditions, such as actual or perceived levels of social support, stressful life events, poverty, and attitudes toward pregnancy and motherhood in general [11-12]. Poor psychiatric healthcare, as a result of society's failure to address mental health issues for women during the postpartum period, appears to be a cause [13].

According to previous studies, the prevalence of PPD ranges from 3% to 69.64%. The differing levels of incidence suggest that some socio-demographic factors are at play. PPD was shown to be prevalent in industrialized nations, such as Singapore, the Netherlands, and Switzerland, with prevalence rates of 3%, 8%, and 11%, respectively [14]. The illness is significantly more common in less developed and resource-constrained nations like Pakistan, India, and Nepal [14-15]. This emphasizes the importance of social position or resource availability in determining the likelihood of postpartum depression in women. In Iran, Chile, South Africa, and Turkey, however, the prevalence of PPD was significantly greater. What is causing the discrepancy across various geographic locations is unknown.

Several socio-demographic risk factors for PPD have been identified, including the link between poverty and low education, which can lead to early marriage. We discovered a larger proportion of postpartum depression in younger women compared to older moms. This might be attributable to tender age since a woman with insufficient experience of caring for a baby or coping with depression is more likely to develop depressive symptoms in the postpartum period [16]. These findings were in line with those of Rich-Edwards, who discovered that women in the youngest age group had nearly double the rate of depression scores as women in other age groups [17]. Housewives with lower incomes were more likely to score higher on the EPDS while Taşdemir et al. [18] and Gumuş et al. [19] both found consistent postpartum depression rates. According to a survey conducted by Dennis et al., poverty is the most important issue for housewives [16].

In addition, we discovered a relationship between the joint family structure and postpartum depression in our research. Those who lived in a shared family system were more likely to suffer from PPD than women who lived in a nuclear family structure. Naveed and Naz came to the same conclusion concerning the combined family setup and the higher incidence of PPD [20]. The importance of social support for new moms cannot be overstated. In other words, women who did not receive any social support were more likely to develop PPD [21]. They found that if women didn't get social support after giving birth, they had a five-fold greater chance of developing postpartum depression, no matter how old they were.

## Conclusions

The prevalence of postpartum depression was found to be relatively high in our study. An increased risk of postpartum depression has been linked to major life events and trauma. There is a favorable relationship between the number of previous children, child health, income status, and family support. The significant prevalence of postpartum depression discovered underscores its importance as a public health concern that necessitates preventative and treatment efforts. The mother's circumstances throughout pregnancy should be known to health providers. They should seek help early in the postpartum period to lessen the risk of depression. As a result, we suggest that mothers be screened following delivery to aid early intervention and management as well as psychosocial support.
